# Chloroform Narcosis

**Published:** 1874-11

**Authors:** 


CHLOROFORM NARCOSIS.—As this is a question which claimed much of my thought and attention some years since, with a view of furnishing additional evidence in favor of that belief, I deem it not inappropriate here to refer to a most interesting case published by myself in 1860, bearing on this very subject, and, so far as I am aware, the only one on record which proves by actual ocular demonstration the truth of this opinion almost beyond a doubt. * * *

During the protracted operation, the patient was placed under the influence of chloroform three or four different times. As the anterior lobes of the brain were fully exposed to view and were terribly lacerated, every opportunity was offered to witness the conduct of the organ under the action of the anæsthetic, and again while free from it. * * * Hemorrhage, which had been free previously, ceased almost entirely after the full anæsthetic impression had been made. It is conceived by the profession that, under the impression of chloroform, the actual vascularity of the brain is increased; that absolute congestion takes place. The present case afforded an ample opportunity to determine this question definitely. Whenever the anæsthetic influence began to subside, the surface of the brain presented a florid and injected appearance. The hemorrhage increased, and the force of the cerebral pulsations became much greater. At these times, so great was the heaving and bulging of the brain that we were compelled to suspend operations until they were quieted by a repetition of the anæsthetic. Then the pulsations would diminish, the cerebral surface recede within the opening of the skull as if by collapse, the organ becoming pale and shrunken with a cessation of the bleeding. In fact, we were convinced that diminished vascularity of the brain was an invariable result of the action of chloroform.

"The changes above referred to recurred sufficiently often during the progress of the operation, in connection with the anæsthetic condition, to satisfy us that there could be no mistake as to cause and effect.

"Again, these changes began invariably to appear with the earliest influence of the agent. As consciousness diminished, in a corresponding ratio there was diminution of vascularity of the brain; and as the mental powers would return, the contrary would occur."

